# Antimicrobial Drug–Selection Markers for *Burkholderia pseudomallei* and *B. mallei*

**DOI:** 10.3201/eid1411.080431

**Published:** 2008-11

**Authors:** Herbert P. Schweizer, Sharon J. Peacock

**Affiliations:** Colorado State University, Fort Collins, Colorado, USA (H.P. Schweizer); Mahidol University, Bangkok, Thailand (S.J. Peacock); University of Oxford, Churchill Hospital, Oxford, UK (S.J. Peacock)

**Keywords:** antimicrobial drug–selection markers, select agents, *Burkholderia pseudomallei*, *Burkholderia mallei*, melioidosis, glanders, perspective

## Abstract

Genetic research into the select agents *Burkholderia pseudomallei* and *B. mallei* is currently hampered by a paucity of approved antimicrobial drug–selection markers. The strict regulations imposed on researchers in the United States but not in other parts of the world lead to discrepancies in practice, hinder distribution of genetically modified strains, and impede progress in the field. Deliberation and decisions regarding alternative selection markers (antimicrobial and nonantimicrobial drugs) by the international community, regulatory authorities, and funding agencies are needed.

## Abstract 2

Genetic research into these select agents is hampered by lack of permitted markers.

Antimicrobial drug–selection markers are essential tools for the bacterial geneticist. Gene deletions created by the insertion of a region of DNA carrying an antimicrobial drug resistance cassette enable geneticists to select mutants by using bacterial agar into which the relevant drug has been incorporated. Choice of an antimicrobial drug–selection marker for a given bacterial species is based on several factors: natural bacterial resistance, markers already available that would work in the organism of interest, and the choice of antimicrobial drugs used to treat natural disease caused by the pathogen.

Select agents are biological agents and toxins that have the potential to pose a severe threat to human health, animal or plant health, or animal or plant products. A recent increase in the number of persons and agencies undertaking research on select agents adds complexity to the use of antimicrobial drug–selection markers. First, there are specific regulations relating to select agents. Second, few genetic tools are available for those select agent organisms that have received limited attention. Third, some species are naturally resistant to a range of antimicrobial drug groups, limiting the choice of drugs for patient care and the possible markers available for experimental studies.

**Restricted Use of Drug-Selection Markers with Select Agents**

In the United States, the acquisition, possession, and use of select agent bacteria are governed by the following Federal Register publications: 42 Code of Federal Regulations (CFR) Parts 72 and 73 for pathogens listed by the Department of Health and Human Services (HHS) and 7 CFR Part 331 and 9 CFR Part 121 for plant and animal pathogens listed by the United States Department of Agriculture (USDA). Some of the agents, including *Burkholderia pseudomallei* and *B. mallei*, are on both the HHS and the USDA lists. Restrictions and regulations include the application of antimicrobial drug–selection markers for genetic manipulation of these bacteria. A person or entity may not conduct a restricted experiment with a select agent unless approved by the appropriate entities. Restricted experiments pertaining to use of selection markers are defined in 42 CFR Part 73, §73.13, section (b)(1), and the National Institutes of Health (NIH) Recombinant DNA Guidelines (Section III-A-1-a) as follows: experiments utilizing recombinant DNA that involve the deliberate transfer of a drug resistance trait to microorganisms that are not known to acquire the trait naturally, if such acquisition could compromise the use of the drug to control disease agents in humans, veterinary medicine, or agriculture. The NIH Office of Biotechnology Activities is responsible for these guidelines. Issues concerning transfer of drug resistance traits to select agents are brought to the Intragovernmental Select Agents and Toxins Technical Advisory Committee for assessment. The final decision is made by HHS, USDA, or both, depending on the agent.

Current policy is that persons or entities must apply for approval for use of even those drugs (antimicrobial and others) for which it has clearly been established that such use will not compromise their ability to control a particular disease agent in humans, veterinary medicine, or agriculture. Even if approval for use of these markers is granted to a number of institutions or persons, such approval does not automatically mean that their use is unrestricted and, therefore, exempt from restrictions under this policy.

**Special Considerations for *B. pseudomallei* and *B. mallei***

*B. pseudomallei* and *B. mallei* are 2 closely related select agents that cause melioidosis and glanders, respectively. *B. mallei* causes natural glanders, a rare disease of equids (*1*), although it can also cause rare infections in humans (*2*). By contrast, the disease caused by *B. pseudomallei,* human melioidosis, is endemic to much of Southeast Asia, northern Australia, and other parts of the Tropics around the world (including Central America and South America) and causes thousands of cases each year (*3*,*4*). These 2 organisms are intrinsically resistant to many antimicrobial drugs, including first-, second-, and third-generation cephalosporins; penicillins; and polymyxin B (*3*). *B. pseudomallei* is naturally resistant to gentamicin, but *B. mallei* is susceptible because of deletion of the genes encoding the AmrAB-OprA efflux pump (*5*). These 2 species are usually susceptible to ceftazidime, the carbapenems, amoxicillin-clavulanate, piperacillin-tazobactam, doxycycline, and trimethoprim-sulfamethoxazole (TMP-SMX) (*6*). First-line treatment for acute human melioidosis is intravenous ceftazidime or a carbapenem for at least 10–14 days, followed by oral TMP-SMX with or without doxycycline for 12–20 weeks (*3*,*6*). For patients who cannot tolerate first-line therapy or for whom this therapy is contraindicated (e.g., children and pregnant women), the choice of oral therapy is amoxicillin-clavulanate. The choice of treatment for human glanders is uncertain because of the rarity of this disease, but clinical experts suggest that treatment should be the same as for *B. pseudomallei*. Acquired resistance to carbapenem drugs has not been reported, and the rate of acquired resistance to ceftazidime and amoxicillin-clavulanate is low (<0.2%) (*6*). Acquired resistance to doxycycline is 2% and to TMP-SMX is geographically variable (2.5% in Australia compared with 13%–16% in northeast Thailand) (*6*).

**Approved Selection Markers for *B. pseudomallei* and *B. mallei***

The markers approved for use with *B. pseudomallei* and *B. mallei* in the United States are kanamycin, gentamicin, zeocin, and polymyxin B (for *B. pseudomallei*), and kanamycin, zeocin, and polymyxin B (for *B. mallei*). Because both species are naturally resistant to polymyxin B, this drug is therefore of little use for the genetic manipulation of these bacteria. *B. pseudomallei* is almost always naturally resistant to gentamicin and other aminoglycosides, although ≈1 in 1,000 isolates cultured from patients with cases of melioidosis at a large hospital in northeast Thailand, where >250 cases are seen each year, were found to be susceptible. *B. mallei* is naturally susceptible to gentamicin, but this marker is prohibited for use with this species because it could potentially be used to treat infection. However, gentamicin can be used as a marker in *B. pseudomallei* if the strain used is naturally susceptible. During 1990–2005, the Wellcome Unit in Thailand identified 4 such strains (708a, 2188a, 2188b, and 3799a) in 3 patients with melioidosis (*7*). One potential drawback is that these strains are poorly characterized. For example, whether these strains are representative of the bacterial population as a whole is not clear.

To answer this question, the Wellcome Unit undertook sequence typing and determined that strain 708a is sequence type (ST) 23, strains 2188a and 2188b are ST47, and strain 3799a is ST154. Because all 3 STs have been previously identified, and several gentamicin-resistant strains have been identified for each of the 3 clones, the susceptible strains are not rare in population genetic terms. Recent unpublished observations from our laboratories indicate that the gentamicin susceptibility in strains 708a, 2188b, and 3799 results from a deletion (708a) or lack of expression (2188b and 3799a) of the *amrAB-oprA* efflux pump operon. These results suggest that 708a may be a natural candidate for genetic manipulation experiments that use gentamicin, spectinomycin, streptomycin (*8*), and nourseothricin (*9*), and validate the use of laboratory-constructed Δ(*amrAB-oprA*) mutants in such experiments.

Kanamycin and zeocin can be used for genetic manipulation of *B. pseudomallei* and *B. mallei*, especially when driven from constitutive promoters; but even then, high concentrations of antimicrobial drugs are required (*10*). The recent development of site-specific recombinase systems for use in *Burkholderia* spp. enables in vivo excision and recycling of selection markers, thus expanding the use of the few precious markers currently approved for genetic manipulation of these bacteria (*10*).

Approval has also been granted for testing of some nonantimicrobial drug–selection markers such as tellurite and triclosan. Unpublished work from our laboratories showed that tellurite resistance conferred by *kilA-telAB* (*11*) may be a useful marker for *B. pseudomallei* (MIC ≈1 μg/mL) and *B. mallei* (MIC <0.5 μg/mL). Similarly, FabL-mediated triclosan resistance (*12*) may be useful in *B. mallei* (MIC = 5 μg/mL) but not in *B. pseudomallei* (MIC >64 μg/mL).

**Restricted Selection Markers**

Consideration of currently restricted selection markers highlights several possible candidates. Until relatively recently, chloramphenicol was used in Thailand for the oral phase of melioidosis treatment, but this use ceased after a clinical trial showed it to be unnecessary (*13*). Rare exceptions to this exist, an example of which is a patient with neurologic involvement who is infected with an organism that is resistant to TMP-SMX or who cannot tolerate this drug. Chloramphenicol penetrates well into the brain; amoxicillin-clavulanate does not. Neurologic involvement occurs infrequently (1.5% of cases in Thailand; *14*), and the chance of neurologic disease with a strain that is resistant to TMP-SMX developing in a patient is low. Use of chloramphenicol for postexposure prophylaxis has not been reported, and its considerable side effects make it a drug of last choice. If this marker were to be allowed, it should never be used in a strain resistant to TMP-SMX. Other potential markers may encode resistance to members of the fluoroquinolone group. These drugs have poor activity in vitro (*6*) and are not recommended for the treatment of melioidosis because of their poor clinical efficacy. A comparison of ciprofloxacin and azithromycin for 12 weeks versus TMP-SMX and doxycycline for 20 weeks demonstrated relapse rates of 22% and 3%, respectively (*15*). The relative contribution of differences in treatment duration to the rates of relapse is not known. However, treatment of 57 adult melioidosis patients with ciprofloxacin or ofloxacin for a median of 15 weeks was associated with an unacceptably high failure rate of 29% (*16*). Therefore, because they are clinically of little use, we reasoned that some of the recently discovered enzyme-mediated fluoroquinolone resistance determinants, e.g., *qnrA* and *aac(6′)-Ib* conferring resistance to ciprofloxacin and norfloxacin (*17*), may be useful for genetic manipulation in *Burkholderia* spp. However, in exploratory experiments they did not confer sufficient levels of norfloxacin resistance to *B. thailandensis* to be of genetic utility (K.-H. Choi and H.P. Schweizer, unpub. data).

**International Discrepancies**

In addition to being limited in range, permissible markers are also subject to considerable international discrepancies in practice and in regulations. For example, although TMP-SMX and doxycycline are first-line drugs for treatment of melioidosis in disease-endemic regions, researchers in other parts of the world consistently use trimethoprim and tetracycline (which leads to cross-resistance with doxycycline) as markers. Use of markers that are prohibited in the United States but not elsewhere leads to several problems. First, mutant strains that are resistant to either agent cannot be imported to and used in the United States, which limits scope for collaborations and sharing of strains. Second, US publications describing select agent research are monitored, and investigators using nonapproved markers risk mandated destruction of their mutant collections containing such markers. Furthermore, this discrepancy encourages US researchers to enlist colleagues abroad to advance their research, although such research cannot be financed by NIH. A possible argument for use of trimethoprim is that natural resistance is seen at a relatively high frequency (at least in isolates from Thailand), and so its use as a marker may be permissible according to the regulatory guidelines. Furthermore, handling of these organisms in a Biosafety Level 3 facility while wearing protective clothing limits exposure risk for workers. However, we consider it inappropriate to create resistance to an antimicrobial drug that is a first-line treatment for melioidosis and that is the agent of choice for postexposure prophylaxis after a laboratory accident in which a worker has had substantial accidental exposure to *B. pseudomallei* or *B. mallei* (*18*).

## Conclusions

The list of approved drugs, antimicrobial and nonantimicrobial, and their respective selection markers is evolving, but its evolution has not been very transparent because no listing of officially approved drugs and allowed selection markers is publicly available. Unlike all other bacterial select agents, no approved attenuated strains of *B. pseudomallei* and *B. mallei* are currently available. Strains of select agents can be excluded from the regulations if the request is accompanied by data showing that the strain is no longer virulent or that the strain is attenuated. Antimicrobial drug–resistance markers, including those used in human and veterinary medicine, can be introduced into excluded strains after approval by local institutional biosafety committees. To our knowledge, no exclusions had been requested of any strain of *B. pseudomallei* and *B. mallei* at the time of the writing of this article. Recent initiatives from the NIH Institute of Allergy and Infectious Diseases regarding funding of research for development of nonantimicrobial drug–selection markers may alleviate some of the problems raised in this article, but not all. Failure to address these issues in a timely manner may compromise genetic research with *B. pseudomallei* and *B. mallei*, cause loss of interest by existing researchers, and contribute to failure to recruit new persons with expertise into the field. We propose that the international community, regulatory authorities, and funding agencies should meet and make timely and conclusive decisions to resolve these problems.
